# Urinary microbiota and bacterial membrane vesicles in chronic kidney disease: contribution to antimicrobial-resistant urinary tract infections

**DOI:** 10.3389/fcimb.2026.1748638

**Published:** 2026-03-03

**Authors:** Naoko Shibata, Ayumi Yoshifuji, Emi Oyama, Motoaki Komatsu, Tatsuhiko Azegami, Kaori Hayashi, Yoshikazu Ishii, Naoki Hasegawa, Ho Namkoong

**Affiliations:** 1 Department of Infectious Diseases, Keio University School of Medicine, Tokyo, Japan; 2 Division of Infectious Diseases and Infection Control, Keio University Hospital, Tokyo, Japan; 3 Clinical Infectious Diseases Center, Keio University Hospital, Tokyo, Japan; 4 Department of Nephrology, Tokyo Saiseikai Central Hospital, Tokyo, Japan; 5 Division of Nephrology, Endocrinology and Metabolism, Department of Internal Medicine, Keio University School of Medicine, Tokyo, Japan; 6 Center for the Planetary Health and Innovation Science (PHIS), The IDEC Institute, Hiroshima University, Higashihiroshima, Japan

**Keywords:** antimicrobial resistance genes, bacterial membrane vesicles, chronic kidney disease, metagenomic analysis, urinary microbiome

## Abstract

Chronic kidney disease (CKD) is associated with an increased risk of severe urinary tract infections (UTIs), particularly those caused by antimicrobial-resistant bacteria. Although urinary microbiota and bacterial membrane vesicles (BMVs) are thought to contribute to UTI pathogenesis, their roles in CKD remain insufficiently understood. In this exploratory study, urine samples were collected from 10 male patients with CKD (eGFR <45 mL/min/1.73 m²) and 10 male non-CKD controls (eGFR ≥60 mL/min/1.73 m²). Urinary microbiota and BMV fractions were isolated and analyzed to compare microbial composition and antimicrobial resistance gene (ARG) profiles, and to evaluate their potential involvement in UTI development and the emergence of antimicrobial resistance in CKD. Both fractions were subjected to shotgun metagenomic sequencing; metagenomic analysis of BMVs was performed using pooled samples within each group. In addition, BMV fractions were characterized by transmission electron microscopy and 16S rRNA gene PCR. Urinary microbiota α-diversity was significantly lower in patients with CKD than in controls (ACE index, *p* = 0.04). Vesicle-like structures consistent with BMVs, with diameters of 20–200 nm, were detected in urine samples from both controls and patients with CKD. Principal coordinate analysis demonstrated that BMV fractions clustered within the corresponding urinary microbiota profiles. Furthermore, multiple antimicrobial resistance genes (ARGs), including *ftsI* and *adeF*, were identified in both urinary microbiota and BMV fractions. This study provides exploratory evidence of reduced urinary microbiota α-diversity in patients with CKD and the presence of ARGs in both urinary microbiota and BMV fractions from controls and patients with CKD. These findings suggest microbiological factors that may contribute to the high incidence of antimicrobial-resistant UTIs in this population. Future validation in larger cohorts with individual-level BMV profiling will be required to determine whether analyses focusing on urinary microbiota and BMVs can contribute to a better understanding of antimicrobial-resistant UTIs and to improved infection risk assessment in patients with CKD.

## 1 Introduction

The prevalence of chronic kidney disease (CKD) is increasing worldwide and is associated not only with progression to end-stage kidney disease, but also with an elevated risk of various complications, including infections and cardiovascular diseases (Jha et al., 2013). Among these, urinary tract infection (UTI) is one of the most common complications in patients with CKD, showing higher recurrence and severity rates than in the general population, and a greater risk of progression to severe outcomes such as sepsis (Chao et al., 2021). Although antimicrobial agents remain the mainstay of UTI treatment, patients are known to have a higher risk of developing antimicrobial-resistant UTIs (Vacaroiu et al., 2022), raising concerns about limited therapeutic options, prolonged infection, and more severe disease courses. To date, explanations for the high incidence, severity, and antimicrobial resistance of UTIs in CKD have primarily focused on host-related factors such as impaired immune function and urinary flow abnormalities (Ishigami and Matsushita, 2019). However, advances in next-generation sequencing technologies have revealed diverse resident urinary microbiota, even in urine previously considered sterile (Wolfe and Brubaker, 2015). Emerging evidence suggests that urinary microbiota contribute to urinary tract homeostasis and defense against infections (Perez-Carrasco et al., 2021). In patients with CKD, factors such as proteinuria, altered urine pH, and impaired voiding function may create environmental conditions that disturb the microbiota balance. However, alterations in the urinary microbiota of patients with CKD and their impact on the development and severity of antimicrobial-resistant UTIs remain poorly understood.

Bacterial membrane vesicles (BMVs) have recently attracted increasing attention in the field of infectious diseases. BMVs are nanoscale structures, approximately 20–300 nm in diameter, that are released into the extracellular environment and can encapsulate a wide range of molecules, including cell wall components, toxins, RNA/DNA, and antibiotic resistance genes (ARGs) (Toyofuku et al., 2019, 2023). These vesicles are implicated not only in enhancing bacterial pathogenicity and modulating host immune responses but also in mediating the horizontal transfer of ARGs, thereby representing a potential mechanism for the dissemination of antimicrobial resistance. However, the composition and molecular content of BMVs in human urine remain unexplored.

In this study, we analyzed urine samples collected from patients with CKD (estimated glomerular filtration rate [eGFR] <45 mL/min/1.73 m², *n =* 10) and non-CKD controls (eGFR ≥60 mL/min/1.73 m², *n =* 10) to separately isolate urinary microbiota and BMVs. We conducted morphological detection of BMVs and shotgun metagenomic analysis of DNA extracted from both the urinary microbiota and BMV fractions. By comparing the bacterial community composition and ARG profiles, this study aimed to elucidate the potential roles of urinary microbiota and BMVs in the increased risk of antimicrobial-resistant UTIs in patients with CKD.

## 2 Materials and methods

### 2.1 Study design and patient population

This study was conducted as a comparative analysis of urinary microbiota and BMV fractions between patients with CKD (eGFR <45 mL/min/1.73 m², *n =* 10) and non-CKD controls (eGFR ≥60 mL/min/1.73 m², *n =* 10), classified according to the KDIGO 2024 Clinical Practice Guideline for Chronic Kidney Disease, with no evidence of kidney damage in the control group (Levey et al., 2012; Stevens et al., 2024). The participants were male patients aged 40–75 years, including outpatients and inpatients at Tokyo Saiseikai Central Hospital and Keio University Hospital, as well as hospital staff members. The presence of comorbidities was not considered an exclusion criterion. The clinical characteristics of the participants are summarized in **Supplementary Table 1**.

### 2.2 Urine sample collection and fractionation

Midstream urine was aseptically collected from all participants (Perez-Carrasco et al., 2021). After collection, samples were promptly transported at 4 °C and stored at 4 °C until processing. Urine was first centrifuged at 3,000 × g for 10 min to separate the pellet from the supernatant. The pellet was retained as the urinary microbiota fraction from which DNA was extracted for metagenomic analysis. The BMV fraction was collected as described previously (Castillo-Romero et al., 2023). Next, the supernatant was centrifuged again at 3,000 × g for 10 min, passed through a 0.22 µm membrane filter (Millex-HP; Merck Millipore, Germany) to remove large particles, followed by concentration using Amicon Ultra-15 centrifugal filter units with a 100-kDa cutoff (Merck Millipore, Germany). To remove cell-free DNA and non-membranous components, the recovered BMV fraction was washed with either 0.1 mol/L cacodylate buffer (pH 7.4; FUJIFILM Wako Pure Chemical Corporation, Japan) for transmission electron microscopy (TEM) or PBS for DNA extraction. Washed BMVs were used for either TEM or DNA extraction, followed by PCR amplification of the bacterial 16S rRNA gene (V3–V4 region) (Klindworth et al., 2013) and shotgun metagenomic analysis. Because the BMV fraction yielded limited amounts of DNA, samples were pooled within each group (control and CKD) and processed as one composite sample per group for shotgun metagenomic sequencing, using a pooled, exploratory design to characterize group-level patterns rather than inter-individual variability.

### 2.3 TEM

BMV fractions were deposited onto carbon-coated copper grids (Nisshin EM Co., Ltd., Japan), negatively stained with 1% aqueous uranyl acetate, and imaged using TEM (JEM-1400Plus; JEOL Ltd., Japan). The vesicle morphology and size were evaluated using the acquired micrographs.

### 2.4 DNA extraction and metagenomic analysis

DNA was extracted from the urinary microbiota and BMV fractions using the QIAGEN DNeasy Plant Mini Kit (QIAGEN, Germany), which allows efficient DNA recovery from urine samples with rigid cell wall structures, including Gram-positive bacteria, according to the manufacturer’s instructions. Using the extracted DNA as a template, the bacterial 16S rRNA gene (V3–V4 region) was amplified using PCR to confirm the presence of bacterial DNA in the BMV fraction. The primer set was selected as previously described (Klindworth et al., 2013). The full primer sequences were as follows: Forward, 5’ -TCGTCGGCAGCGTCAGATGTGTATAAGAGACAGCCTACGGGNGGCWGCAG-3’; Reverse, 5’ -GTCTCGTGGGCTCGGAGATGTGTATAAGAGACAGGACTACHVGGGTATCTAATCC-3’. The PCR products were resolved on a 2% agarose gel to confirm an amplicon of 550 bp, thereby verifying the bacterial origin of the DNA in the BMV fraction, as previously described (Klindworth et al., 2013). For shotgun metagenomics, sequencing libraries were prepared from the extracted DNA by end repair, A-tailing, adapter ligation, and cleanup, followed by sequencing on an Illumina platform with paired-end 150-bp reads (Illumina, USA). Raw reads were quality-filtered and adapter-trimmed using fastp (v0.23.1). Read pairs were discarded if either read contained adapter contamination, more than 10% ambiguous nucleotides (N), or more than 50% low-quality bases (Phred quality score < 5). Next, host-derived sequences were removed by mapping reads to the human reference genome using Bowtie2 (v2.5.4), and assemblies were generated using MEGAHIT. Scaftigs (≥500 bp) were subjected to ORF prediction with MetaGeneMark and de-replication with CD-HIT to construct a nonredundant gene catalog. Taxonomic annotation was performed by aligning the predicted proteins to the microbial subset of the NCBI NR database using DIAMOND. Taxonomic ranks (kingdom, phylum, class, order, family, genus, and species) were assigned using the LCA algorithm. Based on these annotations, relative abundances were calculated for each sample, and α-diversity and β-diversity were assessed. For ARG analysis, non-redundant gene sequences were aligned to the Comprehensive Antibiotic Resistance Database (CARD, version 3.2.6) using the Resistance Gene Identifier (v6.0.2), and individual ARGs were identified. The relative abundance of each ARG was calculated as its proportion of each ARG relative to the total number of ARGs detected in each sample. Per-sample sequencing output before and after quality control is provided in **Supplementary Table 2**. The taxonomic and functional abundance matrices used in this study are provided as **Supplementary Datas 1**, **2**, respectively.

### 2.5 Statistical analysis

α-diversity was evaluated using the abundance-based coverage estimator (ACE), Shannon, and Simpson indices. β-diversity was visualized by principal coordinates analysis (PCoA) based on Bray–Curtis distances. For comparisons between the control and CKD groups, Student’s *t*-test was used to compare α-diversity, and PERMANOVA was applied to β-diversity, reporting pseudo-*F* and *p*-values. The standardized distance was computed as the squared Mahalanobis distance based on the covariance matrix of the PCoA coordinates, and was assessed against the 95% chi-square critical value (*df* = 2; χ^2^ = 5.99). Statistical significance was defined as *p* < 0.05.

### 2.6 Ethical approval

This study was approved by the Ethics Committee of Tokyo Saiseikai Central Hospital (approval number: 2022-017; https://www.saichu.jp/about-division/clinical-trial-center/jisshichu-kenkyu/) and the Ethics Committee of Keio University School of Medicine (approval number: 20236017; https://www.ctr.med.keio.ac.jp/rinri/record/#list). Informed consent was obtained from all participants for inclusion in the study and the publication of the results.

## 3 Results

### 3.1 Urinary microbiota diversity was significantly decreased in patients with CKD

**Figure 1:**
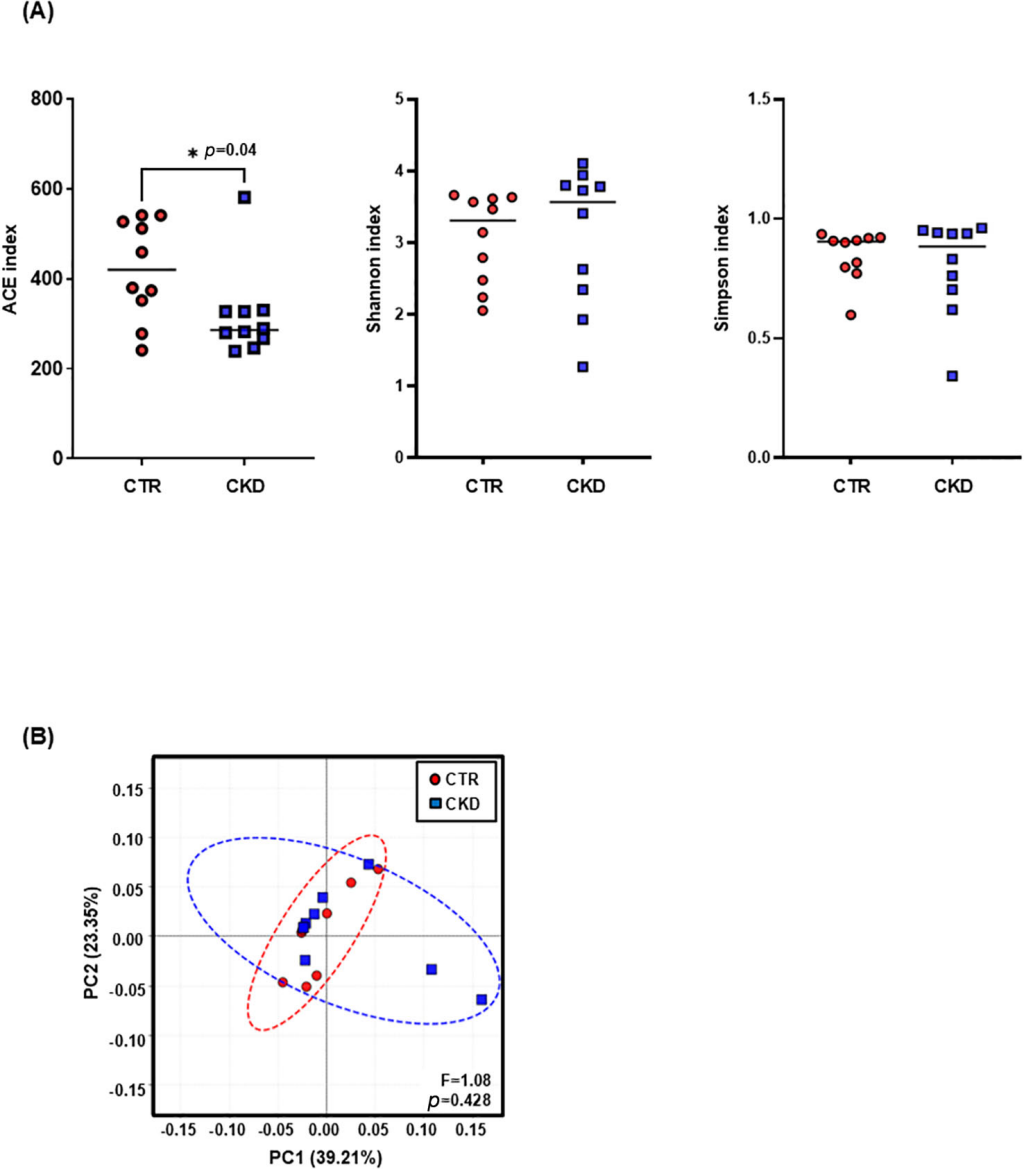
Reduced α-diversity of the urinary microbiota in patients with CKD. Shotgun metagenomic sequencing was performed on DNA extracted from urine samples of controls (*n* = 10) and patients with CKD (*n* = 10). **(A)** α-diversity was assessed using the ACE, Shannon, and Simpson indices. The ACE index was significantly lower in the CKD group compared with controls (*p* = 0.04), whereas no significant differences were observed in the Shannon or Simpson indices. **(B)** PCoA based on β-diversity revealed no clear clustering between the two groups (pseudo-*F* = 1.08, *p* = 0.428). CTR, control group; CKD, CKD group.

Using shotgun metagenomic sequencing, we profiled the composition and diversity of the urinary microbiota at the species level. Taxonomic classification and annotation were performed using the MicroNR database. α-Diversity assessed by the ACE index was significantly lower in the CKD group (316.8 ± 98.4) than in controls (420.5 ± 111.2; *p* = 0.04; **Figure 1**). In contrast, α-diversity assessed using the Shannon and Simpson indices did not differ significantly between the two groups (**Figure 1**). On the other hand, β-diversity evaluated by PCoA on Bray–Curtis distances showed no significant between-group difference (pseudo-*F* = 1.08, *p* = 0.428), and control and CKD samples did not form distinct clusters (**Figure 1B**).

### 3.2 Detection of BMVs in urine

**Figure 2:**
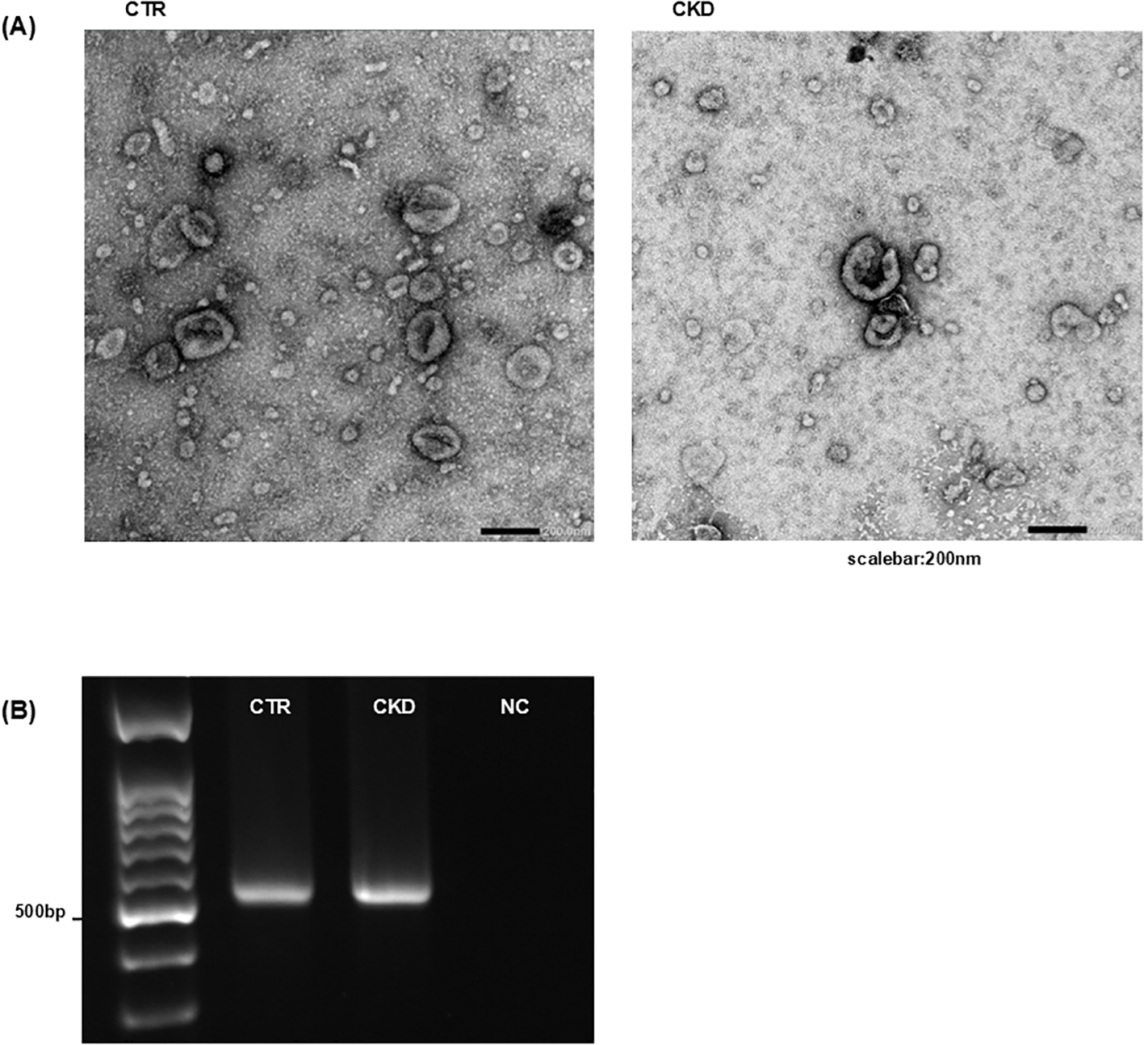
Detection of BMVs in urine from controls and patients with CKD. **(A)** BMV fractions of controls and patients with CKD were negatively stained and visualized by TEM. Numerous spherical vesicles measuring 20–200 nm in diameter were observed in both groups. **(B)** DNA was extracted from the BMV fractions, and PCR targeting the 16S rRNA gene region detected bacterial sequences. CTR, control group; CKD, CKD group. Results shown are representative of multiple independent experiments.

**Figure 3:**
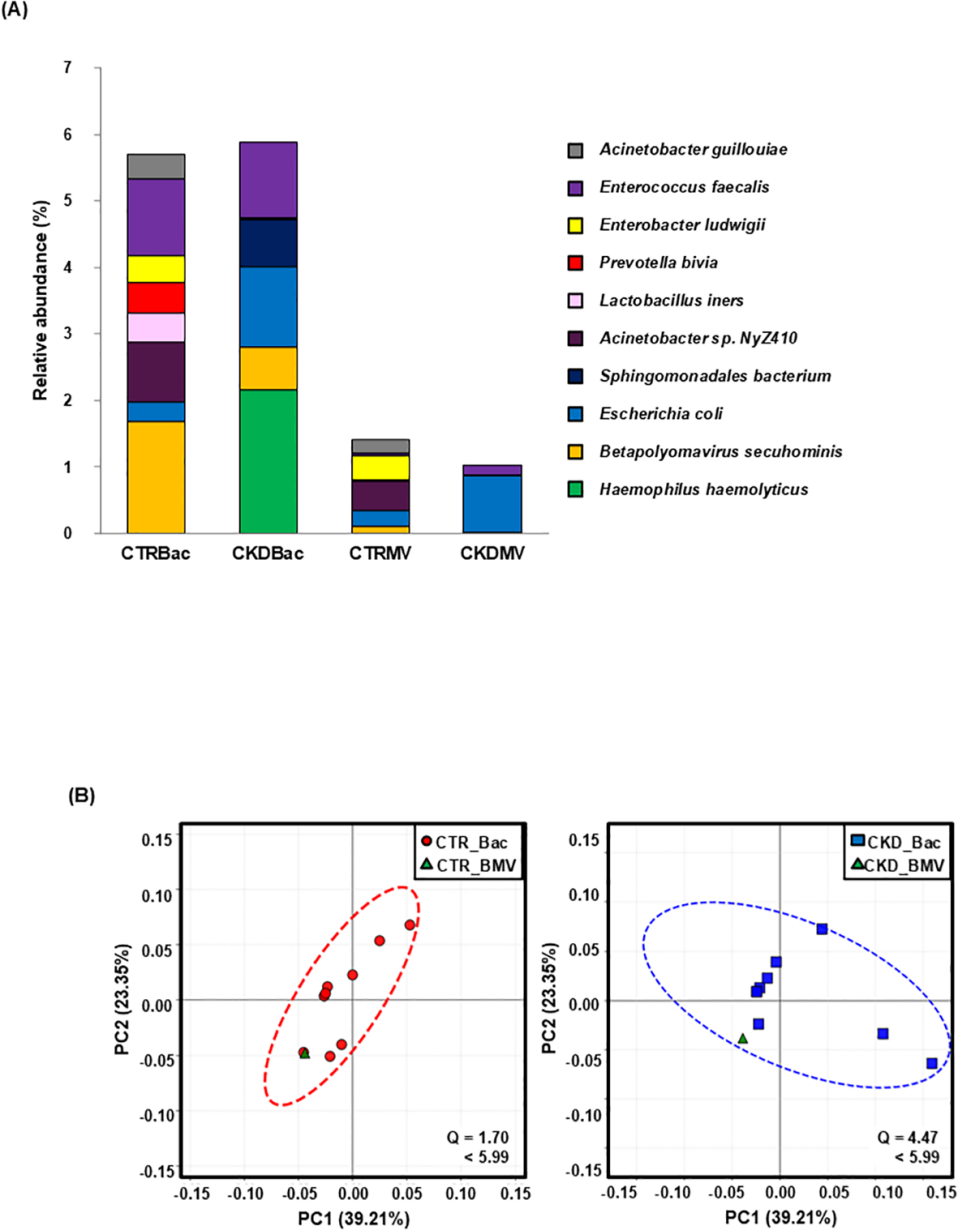
Identification and similarity analysis of microbial DNA in microbiota and BMV fractions. **(A)** DNA extracted from urinary microbiota and BMV fractions isolated from controls (*n* = 10) and patients with CKD (*n* = 10) were subjected to shotgun metagenomic sequencing. The figure shows the relative abundances of the top 10 bacterial species, with bacterial DNA detected in both fractions. **(B)** In PCoA based on gene composition, BMV fractions were located within the 95% confidence ellipses of the corresponding microbiota clusters (standardized distances: control; 1.70, CKD; 4.47, both below the 95% chi-square critical value [*df* = 2; χ^2^ = 5.99]). Each point represents one sample. CTR Bac, microbiota fraction from controls; CKD Bac, microbiota fraction from patients with CKD; CTR BMV, BMV fraction from controls; CKD BMV, BMV fraction from patients with CKD.

TEM revealed numerous spherical vesicles 20–200 nm in diameter in the urine of both controls and patients with CKD (**Figure 2A**). These vesicles possessed membrane structures that were morphologically consistent with BMVs. PCR targeting the V3–V4 region of the 16S rRNA amplified products of the expected size (550 bp) from the BMV fraction (**Figure 2B**), indicating the presence of bacterial DNA. Shotgun metagenomic sequencing identified diverse bacterial taxa in both the urinary microbiota and BMV fractions, including typical urinary commensals such as *Lactobacillus iners*, *Prevotella bivia*, and *Enterococcus faecalis* (Curtiss et al., 2018), which were detected in both controls and patients with CKD (**Figure 3A**). Based on PCoA of gene composition profiles, we calculated the standardized distance of each BMV fraction to the centroid of the corresponding microbiota cluster; the distances were 1.70 for controls and 4.47 for CKD, both below the 95% chi-square critical value (*df* = 2; χ^2^ = 5.99), indicating that BMV fractions in both groups lay within the 95% confidence ellipses of their respective microbiota clusters (**Figure 3B**). To visualize the overlap between the urinary microbiota and BMV fractions, we compared bacterial species detected in each fraction. Venn diagrams showing shared and unique bacterial species in the control and CKD groups are presented in **Supplementary Figure 1**.

### 3.3 Detection of ARGs

**Figure 4:**
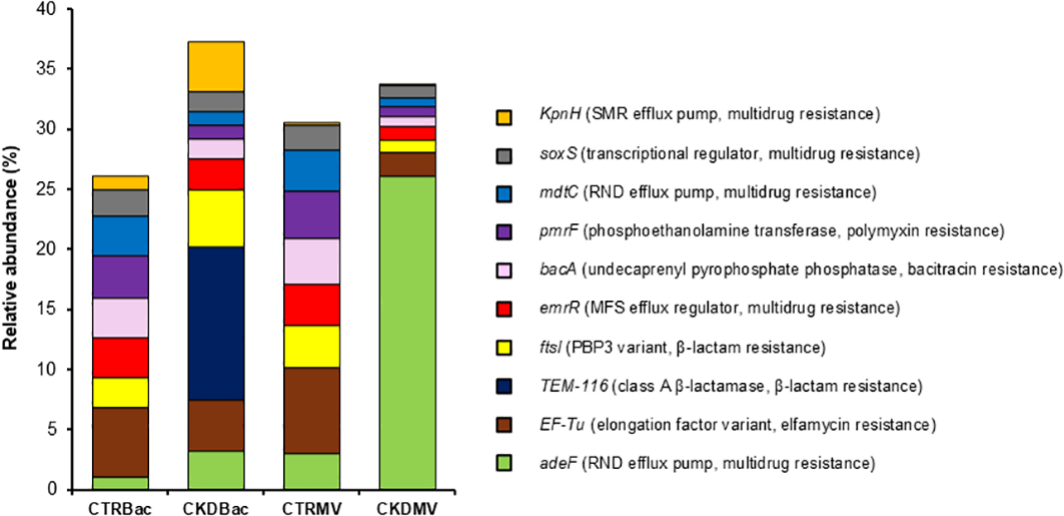
Detection of ARGs in microbiota and BMV fractions. DNA extracted from urinary microbiota and BMV fractions isolated from controls (*n* = 10) and patients with CKD (*n* = 10) were subjected to shotgun metagenomic sequencing, and ARGs were annotated using the CARD database. Multiple ARGs were detected in both fractions from both groups. For relative abundances of ARGs, statistical comparisons were not performed for the BMV fractions because samples were pooled within each group (*n* = 1), and no significant differences were observed between CKD and controls in the microbiota fraction (*p* = 0.61). Figure labels are abbreviated; full CARD annotations are listed below. *KpnH* (small multidrug resistance [SMR] efflux pump, multidrug resistance); *soxS* (transcriptional regulator, multidrug resistance); *mdtC* (RND efflux pump, multidrug resistance); *pmrF* (phosphoethanolamine transferase, polymyxin resistance); *bacA* (undecaprenyl pyrophosphate phosphatase, bacitracin resistance); *emrR* (major facilitator superfamily [MFS] efflux regulator, multidrug resistance); *ftsI* (PBP3 variant, β-lactam resistance); *TEM-116* (class A β-lactamase, β-lactam resistance); *EF-Tu* (elongation factor variant, elfamycin resistance); *adeF* (resistance–nodulation–cell division [RND] efflux pump, multidrug resistance). CTR Bac, microbiota fraction from controls; CKD Bac, microbiota fraction from patients with CKD; CTR BMV, BMV fraction from controls; CKD BMV, BMV fraction from patients with CKD.

Shotgun metagenomic sequencing of the urinary microbiota and BMV fractions, followed by annotation against the CARD, identified multiple ARGs in both fractions from controls and patients with CKD (**Figure 4**). Representative genes included *ftsI* (PBP3 variant, β-lactam resistance) and *adeF* (resistance-nodulation-cell division efflux pump, multidrug resistance) (**Figure 4**). Because the BMV metagenomes were pooled within each group (*n* = 1 per group), no between-group statistical testing was performed for the BMV ARG profiles. In the microbiota fraction, the relative abundances of ARGs did not differ significantly between the control and CKD groups (*p* = 0.61). To further illustrate the distribution of representative ARGs across fractions and groups, a heatmap based on normalized copy numbers is shown in **Supplementary Figure 2**.

## 4 Discussion

Patients with CKD have a higher incidence and severity of UTIs, and are at an increased risk of antimicrobial-resistant UTIs (Vacaroiu et al., 2022). To explore the potential underlying factors, we analyzed urine samples from the perspective of urinary microbiota and BMVs. We found that α-diversity of the urinary microbiota was reduced in patients with CKD. Furthermore, ARGs related to β-lactam antibiotics and multidrug resistance were detected in BMV fractions from urine samples of both controls and patients with CKD.

Although the urinary tract has long been considered sterile, advances in next-generation sequencing technology have enabled the detection of uncultivable resident bacteria, revealing the urinary microbiota (Wolfe and Brubaker, 2015). However, their physiological roles and associations with diseases remain unclear. Most previous studies have focused on women with a higher incidence of UTI and have been complicated by the contamination risk of midstream urine sampling (Perez-Carrasco et al., 2021). In contrast, male urine has a lower risk of contamination; however, studies in men, especially those with CKD, are scarce. Because CKD confers higher rates and severity of antimicrobial-resistant UTIs irrespective of sex, we analyzed the urinary microbiota using midstream urine samples from male patients with CKD.

Metagenomic analysis of the urinary microbiota in controls and patients with CKD demonstrated a significant reduction in α-diversity in patients with CKD when assessed by the ACE index, whereas no significant differences were observed when α-diversity was evaluated using the Shannon or Simpson indices. Because the ACE index primarily reflects species richness, while the Shannon and Simpson indices are more strongly influenced by species evenness, these results indicate that the observed reduction in α-diversity in CKD is mainly attributable to a decrease in species richness rather than to alterations in community evenness.

Resident microbial communities restrain pathogen invasion through interspecies competition; thus, reduced α-diversity indicates a loss of homeostasis that may permit pathogen establishment (Spragge et al., 2023). Studies on intestinal infections have shown that diminished diversity weakens colonization resistance to Salmonella enterica serovar Typhimurium (Spragge et al., 2023) and facilitates the establishment of antimicrobial-resistant bacteria (Shayista et al., 2025). Accordingly, reduced urinary α-diversity in CKD may contribute to the development and severity of antimicrobial-resistant UTIs.

We performed morphological and molecular analyses of urinary BMVs and confirmed the presence of BMV-like vesicles and bacterial DNA in both controls and patients with CKD. PCoA based on genetic profiles showed that genes detected in BMV fractions and urinary microbiota clustered together, supporting their bacterial origin. BMV fractions were prepared under conditions designed to exclude intact bacterial cells and cellular debris by centrifugation and 0.22µm filtration. After ultrafiltration, multiple buffer exchange steps were performed to remove free DNA. In addition, host-derived sequences were removed during metagenomic analysis, supporting the interpretation that the detected genetic material originated from BMVs. However, complete exclusion of host-derived extracellular vesicles, externally associated DNA, or small residual debris cannot be guaranteed. Therefore, BMV-associated findings should be interpreted with appropriate caution. In addition, because BMV metagenomic analyses were performed using pooled samples within each group, this approach may mask inter-individual variability and limit the interpretation of diversity metrics. Pooling may also reduce the sensitivity for detecting low-abundance antimicrobial resistance genes and affect their representativeness. Consequently, downstream analyses based on pooled BMV data should be interpreted reflecting group-level trends rather than individual-level associations, and quantitative correlation analyses between BMVs and urinary microbiota could not be conducted.

BMVs are extracellular vesicles released by gram-negative and gram-positive bacteria, containing DNA, RNA, toxins, enzymes, and ARGs. Only a few studies have detected BMVs in human feces or urine (Park et al., 2018; Lee et al., 2021), and these studies relied on specimens subjected to freeze–thaw cycles, raising concerns about contamination from processing artifacts. In this study, we used freshly collected midstream urine from controls and patients with CKD and processed the samples promptly under refrigeration without freeze–thaw steps to enable more reliable detection of urinary BMVs.

Notably, multiple ARGs were detected in microbiota and BMV fractions of urine samples from both controls and patients with CKD. *In vitro* studies have shown that BMVs can encapsulate ARGs and mediate their horizontal transfer to other bacteria (Bitto et al., 2017); however, to the best of our knowledge, no study has directly isolated BMVs from clinical specimens and confirmed ARGs. The detection of ARGs in BMV fractions from freshly collected urine suggests the possibility of BMVs involvement in the retention and dissemination of ARGs within the human urinary tract. This observation is consistent with hypotheses derived from *in vitro* studies and provides clinical, descriptive evidence supporting the potential role of BMVs in antimicrobial-resistant infections, although direct functional contributions could not be demonstrated in the present study. In addition, we could not confirm whether the detected ARGs conferred functional resistance, or whether they could be horizontally transferred via BMVs. Moreover, comprehensive characterization of BMV components, including mRNA, proteins, and lipids, as well as their interactions with host cells and inflammatory potential, remains an important subject for future studies.

Prior studies have indicated that antimicrobial resistance arises through interacting factors, such as (i) reduced microbiota diversity/dysbiosis, (ii) latent ARG carriage, and (iii) antibiotic-driven selection (Shayista et al., 2025). In this study, we observed decreased α-diversity in the urinary microbiota of patients with CKD and detected ARGs in both microbiota and BMV fractions, suggesting clinical evidence for factors (i) and (ii). Taken together, these findings provide descriptive evidence suggesting a microbiological basis for the increased risk of antimicrobial-resistant UTIs in patients with CKD.

This study has several limitations. First, to minimize contamination associated with midstream urine collection, the analysis was restricted to male participants. Although the risk of infection in CKD has been reported to increase regardless of sex (Yang et al., 2020), this restriction limits the generalizability of our findings.

Second, because of the limited sample size, we were unable to perform multivariable analyses to evaluate the independent effects of age, comorbidities, and medication use with sufficient statistical power. Although urinary microbiota diversity has been reported not to decline markedly with aging (Curtiss et al., 2018), age-related immunosenescence, may influence host–microbe interactions in the urinary tract. In addition, metabolic conditions such as diabetes mellitus and vascular comorbidities including hypertension may alter the urinary tract environment through chronic inflammation, metabolic dysregulation, or altered antimicrobial exposure, potentially affecting both the urinary microbiota and the associated ARG profiles. Furthermore, medication use and a history of UTI (**Supplementary Table 1**) may further shape the urinary microbial and resistome composition.

In subgroup analyses comparing patients with CKD with and without diabetes mellitus, no significant differences in α-diversity were observed (data not shown); however, the limited number of cases precluded robust statistical evaluation. Therefore, the group-level differences observed in this study should be interpreted with caution, and further validation in larger cohorts with sufficient statistical power is warranted.

This study provides exploratory evidence that urinary microbiota α-diversity is reduced in patients with CKD and that ARGs are detectable in both urinary microbiota and BMV fractions. Together, these findings indicate an association between alterations in the urinary microbial environment, BMV-associated genes, and an increased risk of antimicrobial-resistant UTI in CKD. Future validation in larger cohorts will be required to clarify whether analyses focusing on urinary microbiota and BMVs can contribute to antimicrobial-resistant urinary tract infection risk assessment in patients with CKD.

## Data Availability

The raw shotgun metagenomic sequencing data have been deposited in the NCBI Sequence Read Archive (SRA) under BioProject accession number PRJNA1418212 and will be made publicly available upon publication of this article. The data can be accessed at: https://www.ncbi.nlm.nih.gov/bioproject/PRJNA1418212.
