# Supplementary material for: Urinary microbiota and bacterial membrane vesicles in chronic kidney disease: contribution to antimicrobial-resistant urinary tract infections
Source: Front Cell Infect Microbiol. 2026 Mar 3;16:1748638. doi: 10.3389/fcimb.2026.1748638 (PMC12992213; doi:10.3389/fcimb.2026.1748638)
Supplement: Supplementary file 1 [file DataSheet1.pdf]

Supplementary Table 1

Category	Variable	Unit / Format	Control (n = 10)	CKD (n = 10)
Background	Age	years (Med [IQR])	46.9 (42.8–51.8)	71.5 (68.0–74.8)
	BMI	kg/m² (Med [IQR])	25.8 (25.0–27.0)	23.5 (22.6–24.6)
	Antibiotic use within 3months	n (%)	0 (0%)	0 (0%)
	Past medical history of UTI	n (%)	0 (0%)	0 (0%)
Diseases	Diabetes mellitus	n (%)	1 (10%)	5 (50%)
	Hypertension	n (%)	4 (40%)	10 (100%)
	Dyslipidemia	n (%)	3 (30%)	8 (80%)
	Hyperuricemia	n (%)	0 (0%)	6 (60%)
	Rheumatoid diseases	n (%)	2 (20%)	0 (0%)
	Malignant tumor	n (%)	0 (0%)	0 (0%)
	Cerebral infarction	n (%)	0 (0%)	1 (10%)
	Myocardial infarction	n (%)	0 (0%)	2 (20%)
Laboratory data	Serum Alb	g/dL (Med [IQR])	4.6 (4.5–4.6)*	3.9 (3.8–4.1)
	Serum Cre	mg/dL (Med [IQR])	0.9 (0.8–1.0)	3.2 (2.4–4.6)
	eGFR	mL/min/1.73 m² (Med [IQR])	72.5 (65.2–85.8)	16.1 (10.6–22.9)
	Urinary TP/Cr	mg/g Cr (Med [IQR])	— *	1.0 (0.3–2.7)
	HbA1c	% (Med [IQR])	5.6 (5.4–5.8)	6.2 (5.8–6.7)

Med, median; IQR, interquartile range; BMI, body mass index; UTI, urinary tract infection; Alb, serum albumin; Cre, serum creatinine; eGFR, estimated glomerular filtration rate; TP/Cr, urinary total protein-to-creatinine ratio; HbA1c, hemoglobin A1c.
*Data unavailable for the control group.

Supplementary Table 2

SampleID	Raw_Base(G)	Clean_Base(G)
CTR01Bac	8.91	7.2
CTR02Bac	6.79	6.41
CTR03Bac	8.4	7.25
CTR04Bac	6.51	6.3
CTR05Bac	6.33	6.17
CTR06Bac	8.5	8.36
CTR07Bac	8.94	8.64
CTR08Bac	7.17	7.02
CTR09Bac	6.14	6
CTR10Bac	7.73	7.36
CKD01Bac	6.65	6.44
CKD02Bac	6.11	5.95
CKD03Bac	6.57	6.34
CKD04Bac	6.27	6.1
CKD05Bac	7.43	7.15
CKD06Bac	7.03	6.88
CKD07Bac	6.57	6.14
CKD08Bac	6.26	6.09
CKD09Bac	6.93	6.81
CKD10Bac	6.4	6.3
CTRMV	8.44	8.27
CKDMV	7.91	7.73

Per-sample sequencing output before and after quality control.
Raw_Base (G) and Clean_Base (G) indicate the total number of sequenced bases (in gigabases) before and after quality filtering, respectively.
CTR Bac, microbiota fraction from controls; CKD Bac, microbiota fraction from patients with CKD; CTR BMV, BMV fraction from controls; CKD BMV, BMV fraction from patients with CKD.
