# Supplementary figures and images for: Urinary microbiota and bacterial membrane vesicles in chronic kidney disease: contribution to antimicrobial-resistant urinary tract infections
Source: Front Cell Infect Microbiol. 2026 Mar 3;16:1748638. doi: 10.3389/fcimb.2026.1748638 (PMC12992213; doi:10.3389/fcimb.2026.1748638)

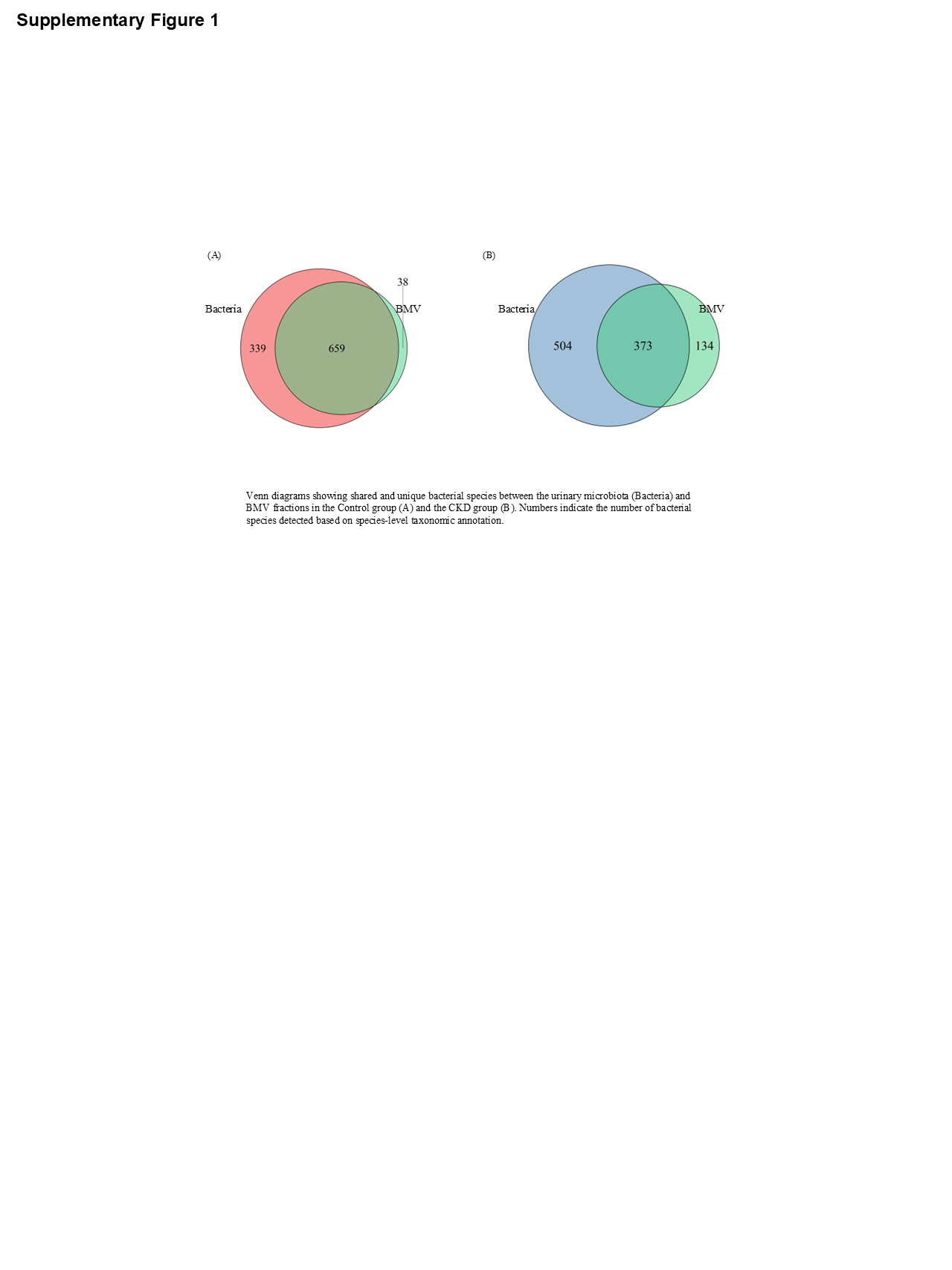

Supplement: Supplementary file 3 [file Image1.tif]

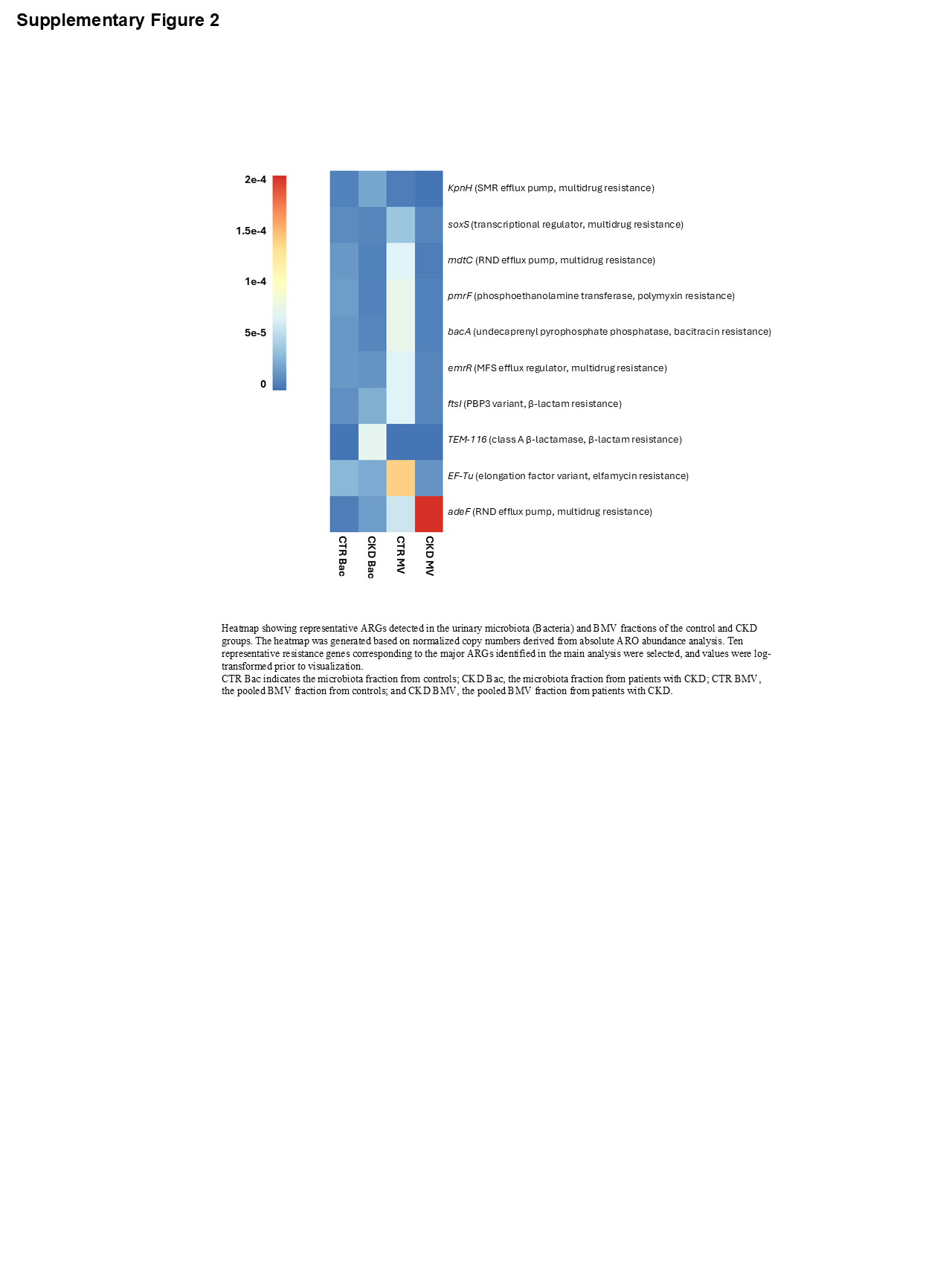

Supplement: Supplementary file 4 [file Image2.tif]
